# Outcomes of post‐prostatectomy radiotherapy at a Regional Cancer Centre

**DOI:** 10.1002/jmrs.240

**Published:** 2017-08-14

**Authors:** Luke Nicholls, Amber Winter, Ashley Harwood, Ashley Plank, Preeti Bagga, Winnie Wong, Eric Khoo

**Affiliations:** ^1^ Radiation Oncology Centres St Andrews Cancer Care Toowoomba Queensland Australia; ^2^ School of Medicine University of Queensland St Lucia, Brisbane Queensland Australia; ^3^ Oncology Research Australia St Andrews Cancer Care Toowoomba Queensland Australia

**Keywords:** Prostate cancer, prostatectomy, radiation therapy, regional cancer centre

## Abstract

**Introduction:**

To investigate the efficacy and toxicity of radiation therapy (RT) after radical prostatectomy (RP) for prostate cancer at Radiation Oncology Centres, Toowoomba.

**Methods:**

The electronic medical records of 130 consecutive patients with histologically proven prostate adenocarcinoma who underwent post‐prostatectomy RT between January 2008 and December 2014 were analysed. Primary endpoint was Biochemical Recurrence (BCR) after RT. BCR was defined by PSA > 0.2 ng/mL and BCR endpoints were analysed using Kaplan–Meier methods. The impact of RT technique and the rates of acute and late toxicities are also reported. Toxicities were graded according to Radiation Therapy Oncology Group (RTOG) criteria.

**Results:**

Median follow‐up time after RT (regardless of technique) was 28 months. BCR occurred in 32 of the 126 patients (25%) whose prostate specific antigen (PSA) levels have been monitored post‐RT. At 24 and 36 months, 85% and 75% of patients were BCR‐free, respectively. Patients with a pre‐RT PSA above 0.2 ng/mL had a higher probability of recurrence than patients with values below 0.2 ng/mL (*P *=* *0.03). RT technique, pelvic nodal irradiation, androgen deprivation therapy, T staging or surgical margin did not significantly impact BCR results.

No patient experienced acute toxicities greater than grade 2. Grade 1 or 2 late gastrointestinal (GI) toxicity occurred in 11% and 1 patient experienced a grade 3 event. 12% of patients developed grade 1 or 2 late genitourinary (GU) toxicity, with evidence of grade 3 severity in only 1 patient. Evidence of a trend in reduction in late GI toxicity with the use of intensity modulated radiation therapy (IMRT) or volumetric modulated arc therapy (VMAT) was apparent but not with late GU toxicity.

**Conclusion:**

At our regional centre, early RT (PSA < 0.2 ng/mL) was associated with significant improvement in BCR‐free survival. Rates of toxicity mirror those of landmark trials which suggest no detriment for our regional prostate cancer patients. The use of IMRT/VMAT techniques was associated with a trend towards reduced rates of GI toxicity.

## Introduction

In the last 10 years, there has been dramatic changes in the pattern of care for men with localised high risk prostate cancer (HRPC). Historically, these men were often managed with androgen deprivation therapy (ADT), external beam radiation therapy (EBRT), or both, while the use of surgery was uncommon due to high risk of occult metastatic disease, concerns of surgical related morbidities and infrequent use of ADT.^1^ Recently, however, the use of radical prostatectomy (RP) in appropriately selected patients has emerged as a viable treatment option due to improved surgical skill, reduction in complication rates^1^ and the increasing use of robotic surgery.^2^ Perhaps there is also unfounded concern from the surgical discipline regarding the toxicity of radiation therapy (RT).^3^ Whilst surgical technique is improving, so too is the delivery of EBRT. Use of intensity‐modulated radiation therapy (IMRT) and image‐guided radiation therapy (IGRT) has markedly reduced the rates of severe gastrointestinal (GI) or genitourinary (GU) toxicity whilst simultaneously allowing dose escalation to the planning target volume (PTV).^4^

Unfortunately, 10–25% of patients experience recurrence after RP.^5^ Patients with adverse risk factors such as high level of prostate‐specific antigen (PSA), stage pT3, positive surgical margins (R1), and Gleason score ≥8 may have up to a 75% chance of biochemical recurrence (BCR) at 10 years, necessitating the use of post‐RP RT. Thus, the increasing utilisation of RP will possibly lead to increased rates of post‐RP RT and increased reliance on regional cancer centres (RCC). The reporting of outcomes from RCC is essential in an era of decentralised medical delivery as the community's expectation is that the quality of treatment received in RCC is equivalent to metropolitan centres.

Toowoomba is classified as an Inner Regional centre and has a population of approximately 150,000.^6^ Radiation Oncology Centres (ROC), Toowoomba provides the sole RT service to a large portion of the Darling Downs and Southwest Queensland regions. This study investigates the efficacy and toxicity of RT after RP for prostate cancer at our RCC.

## Method

This study is predominantly a quality assurance audit and thus a request for waiver of Human Research Ethics Committee review was approved by Oncology Research Australia. The electronic medical records of 130 consecutive patients with histologically proven prostate adenocarcinoma who underwent post‐RP RT between January 2008 and December 2014 were retrospectively analysed. All patients completed the prescribed course of RT and no patients were excluded. All were assessed for Gleason score, TNM stage, surgical margins, PSA (pre‐surgery, pre‐ and post‐RT), RT technique, use of ADT, pelvic nodal irradiation (PNI) and toxicity. Follow‐up time was calculated from the completion of the RT treatment. Radiological staging was performed using computed tomography and technetium‐99 bone scan. Positron‐emission tomography (PET) with gallium‐labelled prostate specific membrane antigen (PSMA) or magnetic resonance imaging (MRI) was ordered at the discretion of the treating clinician.

**Figure 1:**
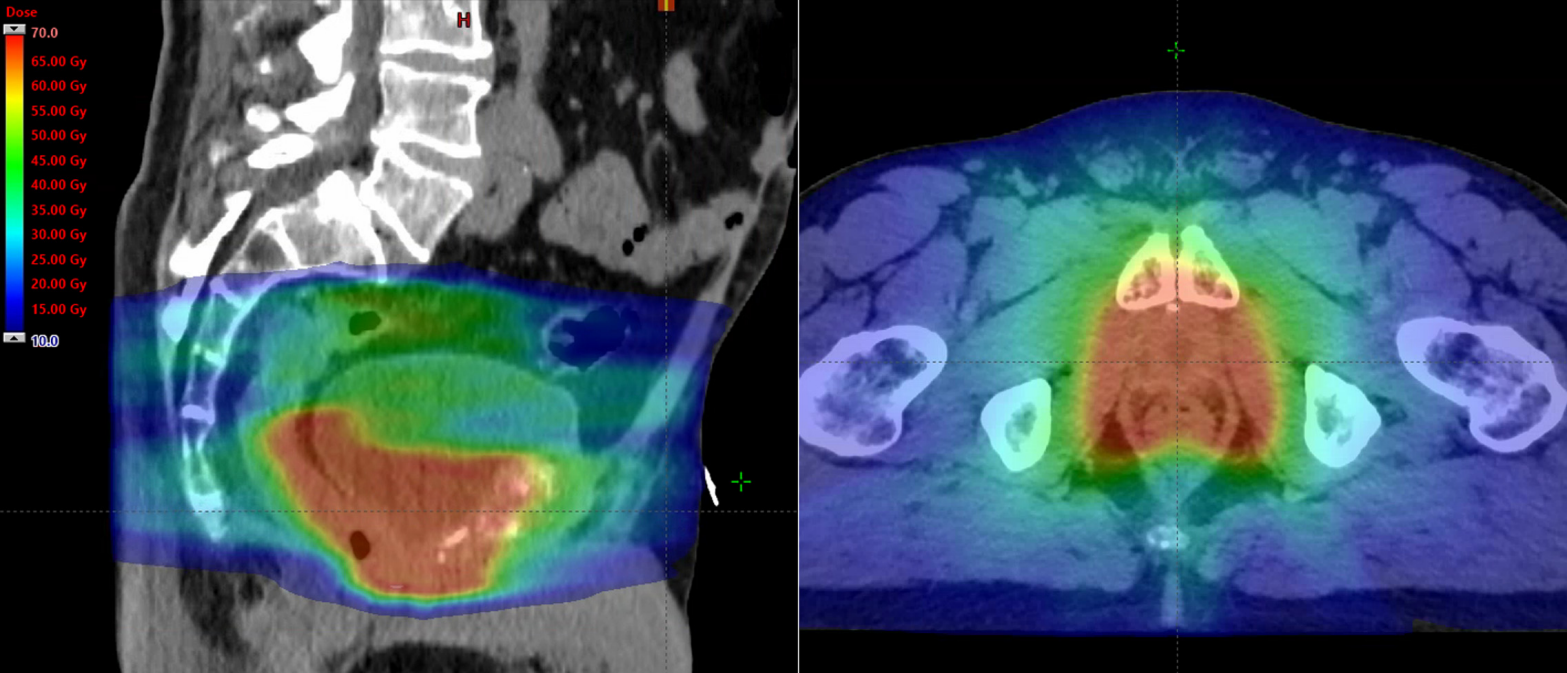
Sagittal and axial views of a typical isodose colour wash (from 10 to 70 Gy) showing the irradiated prostate bed and pelvic nodes using Volumetric Modulated Arc Therapy (VMAT). Dose prescription was 66 Gy in 33 fractions and 52 Gy in 33 fractions, respectively.

The radiation dose and method of delivery varied with time. 3‐dimensional conformal radiation therapy (3D‐CRT) was used exclusively until mid‐2011 whilst IMRT/VMAT techniques were used exclusively from 2012 (see Fig. 1). The clinical target volumes were contoured using the consensus guidelines published by the Australian and New Zealand Radiation Oncology Genito‐Urinary Group.^7^ Pelvic nodes were included in the treatment field at the discretion of the treating physician, primarily based on adverse histological features.

After RT, patients were evaluated by PSA measurements every 3–6 months for at least 5 years. Primary endpoint was BCR after RT. As per RAVES trial protocol,^8^ BCR was defined by PSA > 0.2 ng/mL and BCR endpoints were analysed using Kaplan–Meier methods. The influence of prognostic factors on BCR were assessed using the log‐rank test and Cox regression analysis. *P* values less than 0.05 were considered statistically significant. The secondary endpoint was the assessment of acute and late toxicities graded according to Radiation Therapy Oncology Group (RTOG) criteria.^9^

## Results

Patient characteristics are outlined in Table 1 **.** Median follow‐up time after RT was 28 months (range 1–79) regardless of RT technique. Patients treated with 3DCRT were followed for a median of 29 months (range 3–79) while those treated with IMRT or VMAT had a shorter median follow‐up time of 17 months (range 1–43). Median RT dose was 65 Gy (range 64–72) in 32–38 fractions (median = 33), although a dose of 66 Gy in 33 fraction was used almost exclusively from 2012. The majority of patients had T3 staging (extra‐prostatic extension or seminal vesicle invasion) and Gleason 7 disease was the most common histology. Almost 50% of patients had clear margins at the time of surgery. Mean time to receive RT after RP was 2.64 years. ADT administration was not consistent and was administered in only 28 (22%) patients. The use of prophylactic PNI was similarly sparse and was delivered in 20 (15%) patients.

**Table 1:** Patient characteristics (*n *=* *130)

Age (years)	
Median (range)	64 (49,81)
Tumour stage	
T2	40 (31%)
T3	86 (66%)
Tx	4 (3%)
Nodal status	
N0	91 (70%)
N1	5 (4%)
Nx	34 (26%)
Gleason score	
6	5 (4%)
7	81 (62%)
8	4 (3%)
9	35 (27%)
Not reported	5 (4%)
Surgical margin	
Positive	57 (44%)
Negative	64 (49%)
Equivocal	9 (7%)
Pre‐RT PSA	
≤0.2	96 (74%)
Mean (SD)	0.39 (±0.97)
RT dose (Gy)	
Median (range)	65 (64,72)
Time from RP to RT (years)	
Mean (SD)	2.64 (±2.74)
Technique	
3DCRT	53 (41%)
IMRT or VMAT	77 (59%)
ADT	
Yes	28 (22%)
No	102 (78%)
Pelvic nodal irradiation	
Yes	20 (15%)
No	110 (85%)

RT, radiation therapy; RP, radical prostatectomy; 3D‐CRT, 3D‐conformal radiation therapy; IMRT, intensity‐modulated radiation therapy; VMAT, volumetric modulated arc therapy; ADT, androgen deprivation therapy.

**Figure 2:**
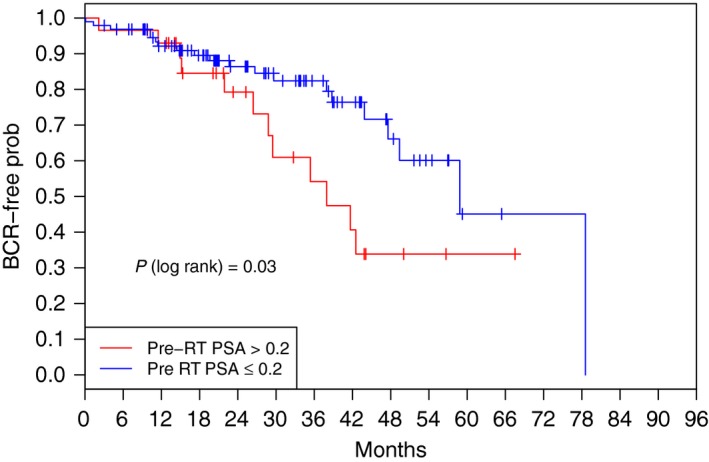
Kaplan–Meier rate estimate of freedom from biochemical recurrence according to pre‐RT PSA level. BCR, biochemical recurrence; RT, radiation therapy; PSA, prostate specific antigen.

BCR occurred in 32 of the 126 patients (25%) whose PSA levels have been monitored post‐RT. At 24 and 36 months, 85% and 75% of patients were BCR‐free, respectively. Patients with a pre‐RT PSA above 0.2 ng/mL had a higher probability of recurrence than patients with values below 0.2 ng/mL (*P *=* *0.03, log rank test) (Fig. 2). RT technique, PNI, androgen deprivation therapy, T staging or surgical margin did not significantly impact BCR results using this method.

Similarly, the impact of age, ADT administration, pre‐RT PSA, inclusion of pelvis irradiation and RT technique on BCR were assessed using Cox regression. Of these five covariates, some evidence exists at the 10% level of significance of dependency between BCR and each of pre‐RT PSA (*P *=* *0.05) and pelvic irradiation (*P *=* *0.08). Specifically for PSA, the risk of biochemical recurrence is estimated to increase by 29% (95% CI: 0–67%) on average for each ng/mL increase in PSA. No systematic effect on BCR of age, administration of ADT, and RT technique is suggested by the data.

**Figure 3:**
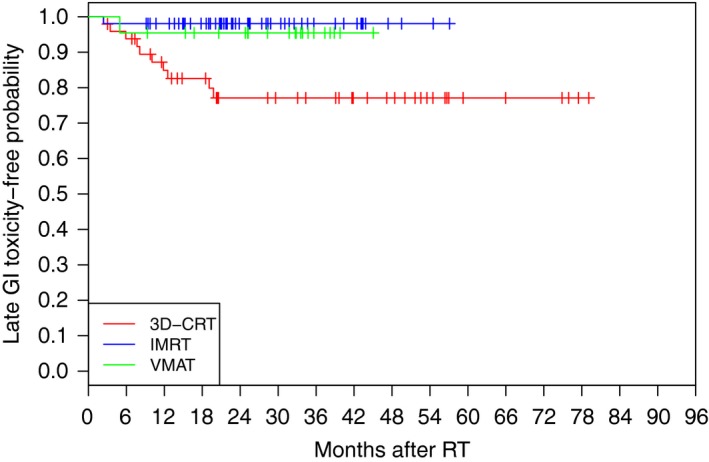
Kaplan–Meier rate estimate of late GI toxicity‐free rate according to RT technique. Median follow‐up time for 3DCRT and IMRT/VMAT patients was 29 months (range 3–79) and 17 months (range 1–43), respectively. Further follow‐up is required to comment on the statistical significance of this relationship.

60 patients (46%) had varying degree of urinary incontinence between surgery and RT. Three patients required a urethral sling procedure prior to RT. RT toxicity is outlined in Table 2. 60% of patients developed grade 1 or 2 acute GI toxicities and 44% developed grade 1 or 2 acute GU toxicities. 11% and 12% of patients developed Grade 1 or 2 late GI and GU toxicity, respectively. 11 patients required colonoscopies or sigmoidoscopies to investigate per rectal bleeding, with only one documented need for photocoagulation of rectal telangiectasia. Similarly, only one patient required surgical intervention for late GU toxicity. There is a trend in the relationship between IMRT/VMAT technique and lower late GI toxicity (*P *=* *0.004) (Fig. 3), however, in light of the short follow‐up (particularly in the patients treated with IMRT/VMAT) these results should be interpreted with caution until a longer follow‐up duration is achieved. No such trend is evident for late GU toxicity (*P *=* *0.72) and further follow‐up is required.

**Table 2:** Rates of acute and late radiation toxicity according to Radiation Therapy Oncology Group (RTOG) criteria. Data include all patients for the current follow‐up period

	Acute toxicity (%)			Late toxicity (%)		
	Grade 0	Grade 1–2	Grade 3–4	Grade 0	Grade 1–2	Grade 3–4
Genitourinary	56	44	0	87.3	12	0.7
Gastrointestinal	40	60	0	88.3	11	0.7

## Discussion

Approximately one‐third of Australians affected by cancer live in regional or rural areas^10^ and those with prostate cancer continue to endure less rates of screening and poorer survival outcomes. This is due to a variety of reasons including lower availability of diagnostic or treatment services, late presentation and diagnosis, lower socioeconomic status, reduced rates of physical activity and a higher proportion of Aboriginal and Torres Strait Islander (ATSI) peoples.^11^, ^12^ The concept of treatment “quality” is also pertinent and has been addressed in the management of head and neck cancer, in which treatment outcomes have been shown to correlate to the volume of cases treated at a particular centre.^13^ Although the management of prostate cancer may not necessarily be as complex as head and neck cancer, regional institutions in Australia are starting to acknowledge the differences in throughput and regularly compare their outcomes to their metropolitan counterparts. ROC Toowoomba delivers approximately 900 courses of radiation treatment annually and offers a full complement of Oncology and Urology services with a fortnightly multidisciplinary meeting. It also has access to advanced imaging modalities including PSMA‐PET and multi parametric MRI (mp MRI).

### Oncologic outcomes

Current national and international guidelines recommend that high risk patients be offered Adjuvant Radiation Therapy (ART)^7^, ^14^, ^15^ after RP due to the evidence provided by three randomised trials^5^, ^16^, ^17^ and a meta‐analysis.^18^ ART is defined as RT commenced prior to 6 months post‐RP. These studies show improved biochemical control, reduced local and metastatic failure, improved quality of life (QoL) with SWOG 8,794^17^ even showing improved overall survival. Whether it be clinician scepticism of benefits or concerns regarding toxicity, the utilisation of ART is lacking, and may be less than 10%.^19^ In this cohort, 16 (12%) patients were treated with ART. This rate is comparable to current national practice, acknowledging the heterogeneous patient population.

The question of ART versus early salvage RT (esRT) upon detectable rising PSA remains to be answered. The available evidence supporting the efficacy of salvage RT is based on retrospective series.^20^, ^21^ Various phase III trials are currently investigating this including the RAVES,^8^ GETUG‐17 (NCT00667069) and RADICALS trials (NCT00541047).

Post‐operative PSA remains a crucial variable in determining the success of esRT^22^ and current guidelines recommend instituting salvage RT at the first sign of PSA recurrence^15^ and PSA < 0.5 ng/mL.^23^ Figure 2 outlines the BCR‐free probability for this cohort, divided into pre‐RT values above or below 0.2 ng/mL. This reveals a significant difference between the two groups, with separation of Kaplan–Meier curves noticeable after only 24 months. This reinforces the importance of close PSA follow‐up in RP patients and the institution of early salvage therapy with PSA < 0.2 ng/mL.

Our 2 and 3 years rates of BCR‐free survival are comparable to landmark Phase 3 trials investigating ART (Table 3), acknowledging a shorter follow‐up duration, differing progression‐free survival (PFS) or BCR definitions and that our cohort includes both adjuvant and salvage patient data.

**Table 3:** A comparison of progression‐free survival (PFS) rates of pivotal phase 3 trials and our institution.^5^, ^16^, ^34^

Study	PFS
SWOG 8794	61% BCR‐free at 5 years
EORTC 22911	74% PFS at 5 years
ARO 9602	80% PFS at 3 years, 56% at 10 years
ROC Toowoomba	85% BCR‐free at 2 years, 75% at 3 years

### Toxicity

The lack of Grade 3 and 4 acute toxicity in our patient population is very reassuring and emphasises excellent tolerance. Other analyses have identified similarly low rates of RTOG grade 3–4 acute GI and GU toxicities, which estimate rates to be less than 3%.^24^

In terms of late toxicity, analyses of multiple cohorts have found approximately 15% rates of RTOG grade 2 rectal toxicity and <5% rates of grade 3 toxicity.^25^ Rates of grade 2 and 3 urinary toxicity are reported to be slightly less (approximately 10% grade 2 and 5% grade 3) in both multi‐ and single‐institution studies.^5^, ^26^ In our series to date, there has only been 1 reported case of Grade 3 late GU and GI toxicity, respectively. In these instances, a cystoscopy was required for urethral strictures and photocoagulation was necessary for bleeding for rectal telangiectasia. Due to the recent adoption of IMRT/VMAT, the median follow‐up of patients treated with these techniques is relatively small at 17 months. Although the use of IMRT/VMAT technique showed a trend towards reducing late GI toxicity, further follow‐up is required to confirm true significance. Nonetheless, the low absolute numbers of severe late GI and GU toxicity are encouraging in our contemporary patient population.

### Androgen deprivation

There is an increasing interest in the use of neoadjuvant and concurrent ADT in the salvage treatment setting, considering its established role in high risk localised prostate cancer.^27^ It has recently been shown that the use of 24 months of concurrent and adjuvant bicalutamide in high risk salvage patients improved overall survival and reduced incidence of metastatic disease.^28^ Even in the era of ultra‐sensitive PSA assays, patients salvaged early (PSA < 0.5) and with negative margins seem to benefit from the addition of ADT.^29^ These results, in addition to the ongoing SPPORT (NCT00567580) and RADICALS (NCT00541047) trials will hopefully clarify the role of ADT in the adjuvant or salvage setting. The use of ADT in our cohort was low (22%) and although there was a trend towards improved BCR, no significant association was found (*P* = 0.15). At our centre, ADT was offered to patients with an adequate performance status, T3 disease (or T2 disease with positive margins) or a high pre‐treatment PSA. This is based on RTOG9601^28^ inclusion criteria.

### Pelvic nodal irradiation

The routine use of prophylactic PNI in addition to prostate bed treatment in high risk patients with biochemical relapse lacks phase III evidence, which has also translated into low utilisation in our series. Retrospective series,^30^ risk stratification formulae^31^ and the emergence of more sophisticated imaging techniques^32^ will continue to guide clinicians in selecting suitable patients for elective PNI.^32^ The SPPORT trial (NCT00567580) is exploring short‐term ADT with or without the addition PNI and RTOG 0924 (NCT01368588) is solely investigating the addition of PNI in unfavourable intermediate or favourable HRPC patients. While waiting for more confirmatory evidence, there seems to be a trend to delivering PNI in the post‐prostatectomy setting, particularly utilising advanced imaging modalities to rule out nodal disease prior to RT. A recent survey of 999 international radiation oncologists^33^ suggested PNI was offered by 74%. There is a similar trend in our own centre since 2015.

### Limitations

This study has inherent limitations as a retrospective, single institution chart review. Ideally, a 5 years median follow‐up would be more clinically meaningful, considering the natural history of prostate cancer. An update of our data in 5 years’ time would allow the reporting of more relevant efficacy end points such as prostate‐specific cancer survival and overall survival as well as more meaningful comments in regards to late toxicity. Next, small sample size from a single institution caries selection biases. For example, some patients with BCR who have particularly high risk disease characteristics may have been offered ADT alone rather than be referred for RT. Finally, while all effort is used to standardise toxicity scoring at our centre, these outcomes are naturally prone to recall and misclassification bias.

## Conclusion

At ROC Toowoomba, early RT (PSA < 0.2 ng/mL) was associated with significant improvement in BCR‐free survival, supporting the role of early RT intervention. The low utilisation of ART corresponds to current national and international practice. Rates of d toxicity mirror those of large landmark trials and suggest no detriment for patients treated at our RCC. The use of IMRT or VMAT techniques was associated with a trend towards reduced rates of GI toxicity. Due to low patient numbers in the ADT and PNI subgroups, their utilisation has not translated to a significant benefit in BCR. This data contributes to the growing evidence of non‐inferior treatment quality delivered in non‐metropolitan centres.
